# Clinical pharmacogenomics in action: design, assessment and implementation of a novel pharmacogenetic panel supporting drug selection for diseases of the central nervous system (CNS)

**DOI:** 10.1186/s12967-021-02816-3

**Published:** 2021-04-15

**Authors:** E. Bothos, E. Ntoumou, K. Kelaidoni, D. Roukas, N. Drakoulis, M. Papasavva, F. A. Karakostis, P. Moulos, K. Karakostis

**Affiliations:** 1 HybridStat Predictive Analytics, Athens, Greece; 2 Institute of Communications and Computer Systems, National Technical University of Athens, Athens, Greece; 3 iDNA Genomics Private Company, Evrota 25, Kifissia, 145 64 Athens, Greece; 4 Department of Psychiatry, Army Hospital (NIMTS), 417 Veterans, 115 21 Athens, Greece; 5 Research Group of Clinical Pharmacology and Pharmacogenomics, Faculty of Pharmacy, School of Health Sciences, National and Kapodistrian University of Athens, Panepistimiopolis, 15771 Zografou, Greece; 6 Paleoanthropology, Senckenberg Centre for Human Evolution and Palaeoenvironment, Department of Geosciences, University of Tübingen, Tübingen, Germany; 7 Institute for Fundamental Biomedical Research, Biomedical Sciences Research Center ‘Alexander Fleming’, 34 Fleming str, 16672 Athens, Vari Greece

**Keywords:** Psychiatry, Diseases of the central nervous system, Cytochromes, Drug metabolization, Adverse drug events, Companion diagnostics, DNA test, Precision medicine and personalized therapy, Dose adjustment, Clinical pharmacogenomics

## Abstract

**Background:**

Pharmacogenomics describes the link between gene variations (polymorphisms) and drug responses. In view of the implementation of precision medicine in personalized healthcare, pharmacogenetic tests have recently been introduced in the clinical practice. However, the translational aspects of such tests have been limited due to the lack of robust population-based evidence.

**Materials:**

In this paper we present a novel pharmacogenetic panel (iDNA Genomics-PGx–CNS or PGx–CNS), consisting of 24 single nucleotide polymorphisms (SNPs) on 13 genes involved in the signaling or/and the metabolism of 28 approved drugs currently administered to treat diseases of the Central Nervous System (CNS). We have tested the PGx–CNS panel on 501 patient-derived DNA samples from a southeastern European population and applied biostatistical analyses on the pharmacogenetic associations involving drug selection, dosing and the risk of adverse drug events (ADEs).

**Results:**

Results reveal the occurrences of each SNP in the sample and a strong correlation with the European population. Nonlinear principal component analysis strongly indicates co-occurrences of certain variants. The metabolization efficiency (poor, intermediate, extensive, ultra-rapid) and the frequency of clinical useful pharmacogenetic, associations in the population (drug relevance), are also described, along with four exemplar clinical cases illustrating the strong potential of the PGx–CNS panel, as a companion diagnostic assay. It is noted that pharmacogenetic associations involving copy number variations (CNVs) or the HLA gene were not included in this analysis.

**Conclusions:**

Overall, results illustrate that the PGx–CNS panel is a valuable tool supporting therapeutic medical decisions, urging its broad clinical implementation.

**Supplementary Information:**

The online version contains supplementary material available at 10.1186/s12967-021-02816-3.

## Background

The variability in drug response with respect to dosing efficacy and planning, as well as potentially controversial drug reactions, have long been considered as limiting factors to the overall improvement of patients’ well-being in many medical domains including psychiatry and neurology [1–6]. It has been long known that the response and efficacy of a drug can be influenced by genes and genetic polymorphisms involved in signaling mechanisms linked to the metabolization of the drug [7–9], and by specific DNA variations, which may impact the activity of encoded proteins and the mechanisms of action of a drug. The study of genes and genetic polymorphisms that influence the variability in drug response and efficacy is known as Pharmacogenetic and Pharmacogenomic (PGx) analysis. Related studies employ methodologies from the fields of molecular, cellular biology and biochemistry, and include clinical statistical analyses on available genomic data, aiming to identify how specific genetic variations entailing altered protein structural features and activities may lead to diverse drug related phenotypic outcomes.

Even though the PGx research is ongoing and novel evidence-based findings are being constantly improved, enriched and accumulated from genomic and clinical studies [10–16], current evidence on sets of important pharmacogenes show that gene-drug associations can be used as clinically valuable predictive PGx markers [10]. The clinical implementation of PGx has been facilitated by the improvement of molecular techniques and methodologies of high sensitivity and specificity, such as genotyping by real time PCR, which can determine a panel of targeted variations on the DNA, in a cost-efficient and time-efficient manner. By exploiting the available PGx data and correlating this information with a patient’s genetic profile, tailored and personalized drug treatments can be determined. Thus, the optimization and personalization of drug treatments by exploiting the available vast genomic and PGx information is more promising than ever before and rapidly becomes integrated into mainstream clinical care strategies [17, 18]. However, selecting a set of gene-drug associations from the vast pool of the available genomic and PGx data for clinical implementation, remains a challenging task.

The identification of variants affecting gene-drug associations is well-documented in the literature and several knowledge bases and databases have been assembled to resource clinical information including clinically actionable gene-drug associations and genotype–phenotype relationships, as well as clinical guidelines and drug labels. A fine example is the PharmGKB (PharmG Knowledge Base) [10], which collects, curates and disseminates published findings and bibliographic evidence supporting associations and relationships between genetic variations and drug responses.

The selection of clinically valuable PGx associations, destined to be used as companion diagnostics, is a demanding task that requires two main criteria: (a) Each gene to be selected due to its participation/role in cell signaling pathways linked to the metabolization or action mechanism of a given drug; and (b) Each clinical annotation and gene variant-drug association to be well-documented in several studies describing positive versus negative results, p-values and study sizes [19], thus providing with an adequate level of validity. An additional selection factor for the development of robust minimal PGx panels is the occurrence of each variation in the population. Variations of high or intermediate occurrences are preferred over variations of extremely rare occurrences. Other databases similarly collect and curate variant-drug associations (ie CPIC [7], SNPedia [20], Drugbank [21]). The selection is based according to the targeted disease/disorder and the drugs of interest.

Over 150 drugs with PGx information related to drug safety are listed on the United States Food and Drug Administration (US FDA); approximately 15% of which fall under the medical field of psychiatry [22]. Psychiatry and Neurology involve conditions like depression, bipolar, schizophrenia, binge eating and more disorders of the central nervous system (CNS) [23], also comprising a set of complicated disorders affecting the mood, the memory and the mobility, including epilepsy, multiple sclerosis and neurodegenerative disorders [24]. Pharmacologic intervention employs several drugs for the treatment of CNS disorders to target specific enzymes, proteins and pathways implicated in the disease. For example, drugs for the management of depression (i.e. escitalopram, sertaline, venlafaxine, and duloxetine), are used to modify receptor interactions (serotonin and norepinephrine re-uptake inhibitors) [25, 26]. Currently known markers of enhanced gene-drug and variant-drug associations for CNS drugs, encompass enzyme-coding genes like the cytochrome P450 family (*CYP*) and the *UGT2B7* (catalyst/metabolic activity); as well as proteins involved in signal transduction, like UGT2B7, EPHX1, FKBP5, ANKK1/DRD2, DRD3, HTR2C, MC4R, GRIK1 and SCN1A (protein channels, receptors, post-translational modifications, linked to both drug dosage and ADEs) [10]. In particular, the cytochrome P450 family, comprising the CYP2C9, CYP2C19 and CYP2D6 enzymes, is known to catalyze the metabolization of some 50% of all approved drugs, including several drugs used in the treatment of CNS diseases. Their star allelic forms are linked to drug-dependent altered metabolization efficiency [27]. Most gene-drug associations are influenced by single nucleotide polymorphisms (SNPs) on the gene and a set of unique SNPs can be used to address a gene-drug association with phenotypical/functional implications. As an example, the rs17782313 variant of the Melanocortin 4 receptor gene (*MC4R*) is directly linked to an increased risk of elevated body weight during treatment with quetiapine [28, 29]. However, in the case of the CYP family, PGx associations have been studied between the star allelic forms rather than unique SNP targets. The star allelic forms can be determined indirectly, via the identification of a combination of SNPs specifically occurring in a unique star allele form. As an example, the *CYP2C9* star alleles *2 and *3 can be identified by the SNP rs1799853 and rs1057910, respectively. The homozygous CYP2C9*1 is an extensive drug metabolizer; while homozygous CYP2C9*2 and *3 are considered as poor metabolizers [10, 30, 31]. A heterozygous genotype of CYP2C9*1/*2 or *1/*3 results in an intermediate metabolization activity of the anti-epileptics valproic acid and phenytoin, indicating a linear gene dose effect [32, 33]. Similarly, the SNPs rs4244285 and rs4986893 indicate the *CYP2C19* star alleles *2 and *3, respectively; while a combination of the SNPs rs28399504 and rs12248560 is required for the determination of the star alleles *4 and *17 [10]. In the case of *CYP2D6*, the SNPs rs35742686, rs5030655, rs5030656 and rs28371725, determine the star alleles *3, *6, *9 and *41, respectively; while for the determination between *4 and *10, the rs3892097 and rs1065852 are required. More information can be found on the respective allele functionality tables of PharmGKB.^1^^,^^2^^,^^3^

In this paper, we present a novel PGx panel of polymorphic targets, used as markers for the determination of personalized drug treatments against disorders of the CNS. We describe the design and selection criteria of the targets of the PGx-CNS panel, and provide the assessment of the linked PGx associations, analysing genotypic and PGx results from 501 CNS patient-derived samples from southeastern Europe (Greece). In addition, we present statistical evidence on population occurrences of each SNP and we analyse the frequencies of clinically useful pharacogenetic conclusions, such as the metabolization activities. Moreover, we provide four exemplar clinical cases illustrating the strong potential of the PGx–CNS panel, as a companion diagnostic assay. Overall, our results illustrate that the PGx–CNS panel is a valuable tool supporting therapeutic medical decisions, urging its broad clinical implementation.

## Materials and methods

### Selection of clinically relevant PGx targets

For the construction of the proposed PGx–CNS panel, twenty-eight approved drugs used for the treatment of diseases of the CNS were selected on the basis of their availability and usage in the European market (Additional file 1). Each drug was used for literature search/mining (PubMed) and knowledge-base (KB) mining on several genomic platforms including the PharmGKB [10], for the identification of known evidence-based gene-drug and variant-drug associations. Subsequently, a subset of the identified targets was selected based on bibliographic findings and drug specificity (Additional file 1). Twenty-four targets were selected and introduced to an *in-house* database, to constitute the PGx–CNS panel, including the variant-drug PGx associations derived by curated knowledge-base of PharmGKB [10]. This set of information was used to form an *in-house* PGx–CNS database comprising the respective PGx associations. The selected targeted genes encode several types of functional proteins, varying from receptors, enzymes, ion channels, and signal transducers (Additional file 2). The assessment and the evaluation of the derived PGx information is described in the Results section.

## Collection of samples—Patients

For the assessment of the PGx–CNS panel human buccal cells were collected from 501 individuals of random age and sex. All individuals are patients diagnosed with one or more conditions in the spectrum of the central nervous system and require medical interventions and prescription with antipsychotic, anti-depressants, anti-epileptic or other drugs analysed here. The collection was performed using Forensic swabs L, DNA-free, ETO-sterilized (Sarstedt, Germany). Each sample was diluted in 500 μl of PBS. The DNA from buccal cell lysates was purified with Purelink genomic DNA mini kit (Invitrogen by Thermo Fischer Scientific). Each DNA sample was quantified by NanoDrop One Spectrophotometer (Thermo Fischer Scientific). The quality control required all samples to be above 30 ng/μl with an A260/A280 ratio of around ~ 1.8 and an A260/A230 ratio within the range of 2.0–2.2. Samples were stored at − 20 °C until further use.

## Genotyping

Genotyping on 501 samples was performed using the TaqMan Drug Metabolism and Custom Genotyping Assays at the QuantStudio™ 12 K Flex Real-Time PCR System (Applied Biosystems by Life Technologies). The PGx–CNS panel included 24 targets. The corresponding sets of primers and probes were purchased by ThermoFischer Scientific. Probe IDs and corresponding genes and SNPs are listed in Additional file 1. The genotyping method was performed following the manufacturer's instructions. Briefly, 25 ng genomic DNA was used in 5 μL reaction volume, including 2.5 μl TaqMan Genotyping Master Mix (Applied Biosystems) and 2.5 μl genomic DNA that was dispensed to each well of 384 well plates. The temperature protocol (95 °C for 15′′ and 60 °C for 85′ʹ for 60 cycles) was performed in accordance with the manufacturer’s instructions. Negative controls did not include DNA and yielded no amplification signal. Raw data were collected from the instrument and analyzed with the QuantStudio 12 K Flex software and the TaqMan Express software, by the method of allelic discrimination. The genotypic data were used for the bioinformatic variant analysis and PGx interpretation.

## Statistical and bioinformatic analysis

The genotyping results were used for the statistical analyses describing the occurrences and their comparison to the frequencies in the population groups: European (commonly CEU: 180 samples of Northern and Western European ancestry); African (commonly YRI: 180 samples of Yoruba in Ibadan, Nigeria) and East Asian (commonly CHB: 90 samples of Han Chinese in Beijing, China); obtained from the publicly available genomic database published in the National Center for Biotechnology Information (NCBI) (dbSNP, www.ncbi.nlm.nih.gov/snp/ and SNPedia, www.snpedia.com/) [34]. Frequency values are available on https://www.ncbi.nlm.nih.gov/snp/ and https://www.snpedia.com/: [34, 35]. The software GraphPad Prism was used for the calculation of the averages, error, and standard deviations. Additionally, the association between population group and gene variant frequencies was statistically evaluated for each of the 24 variants, through a series of Pearson’s chi-square (*χ2*) tests [36]. For each variant, three pairwise comparisons were performed (Table 1, Additional file 3). The strength of each association was determined based on the Cramer’s V coefficient [37]. Given that all tests involved two degrees of freedom, the resulting coefficients were interpreted as weak (0.7–0.20), moderate (0.21–0.34), or strong (0.35 or above) [37]. The Pearson’s Chi-square test assumes that at least 80% of the expected counts are five or more, while none of the individual expected counts are zero [36, 38]. In cases where these sampling adequacy assumptions were violated (i.e., 15 of the 72 comparisons performed**)**, we further confirmed the significance of the findings using the Fisher’s exact test of independence, in agreement with standard statistical recommendations. Given that multiple comparisons were performed (Table 1), the resulting p-values were corrected using the Holm-Bonferroni procedure [39].

**Table 1:** Variant frequency associations and p-values

Gene variants	PGx–CNS-CEU	PGx–CNS-AFR	PGx–CNS-CHB
rs1799853	0.09 (0.08)	**< 0.01 (0.23) **	0.11 (0.09)
rs1057910	0.09 (0.08)	**< 0.01 (0.22) **	0.11 (0.09)
rs4244285	0.02 (0.11)	0.31 (0.06)	**< 0.01 (0.22) **
rs4986893	0.54 (0.03)*	0.60 (0.01)	< 0.01 (0.27)*
rs1051740	0.46 (0.05)	**< 0.01 (0.27) **	0.01 (0.13)
rs1065852	0.01 (0.11)	< 0.01 (0.14)	**< 0.01 (0.35) **
rs12248560	0.01 (0.11)	0.03 (0.10)	**< 0.01 (0.26) **
rs1414334	0.30 (0.06)	**< 0.01 (0.48) **	0.01 (0.15)
rs17782313	0.06 (0.09)	0.12 (0.08)	0.39 (0.06)
rs1799978	0.55 (0.08)*	**< 0.01 (0.15) **	**< 0.01 (0.17) **
rs1800497	0.22 (0.07)	**< 0.01 (0.36) **	**< 0.01 (0.32) **
rs2234922	0.74 (0.03)	**< 0.01 (0.27) **	< 0.01 (0.14)
rs2832407	0.03 (0.10)	**< 0.01 (0.73) **	**< 0.01 (0.25) **
rs28371725	0.35 (0.06)	**< 0.01 (0.22) **	0.01 (0.13)
rs28399504	0.12 (0.04)*	0.69 (0.05)*	> 0.99 (0.04)*
rs35742686	0.17 (0.08)*	0.03 (0.09)*	0.13 (0.08)*
rs3812718	0.41 (0.05)	**< 0.01 (0.28) **	0.75 (0.03)
rs3892097	0.40 (0.05)	**< 0.01 (0.18) **	**< 0.01 (0.24) **
rs4713916	0.34 (0.06)	**< 0.01 (0.33) **	0.08 (0.09)
rs489693	0.01 (0.13)	**< 0.01 (0.32) **	< 0.01 (0.14)
rs5030655	0.59 (0.01)*	0.59 (0.01)*	0.44 (0.04)
rs5030656	0.36 (0.03)*	0.59 (0.01)*	0.44 (0.04)*
rs7668258	0.43 (0.05)	**< 0.01 (0.31) **	< 0.01 (0.14)*
rs963468	0.75 (0.03)	**< 0.01 (0.49) **	0.60 (0.04)

Statistical results of the Chi-square pairwise comparisons of gene variant frequencies between the sample of this study (PGx–CNS, SEC) and the following populations: European CEU (commonly CEU: Northern and Western European ancestry); African, AFR (commonly YRI: Yoruba in Ibadan, Nigeria) and East Asian, CHB (commonly CHB: Han Chinese in Beijing, China). The presented output for each test includes its p-value and the corresponding Cramer’s V coefficient (in parenthesis). The comparisons for which the Fisher’s exact test of independence was additionally used (see “Materials and methods”) are indicated with an asterisk. The p-values in bold are those that remained significant (p-value < 0.01) after adjustment using the Holm-Bonferroni procedure. The degrees of freedom for all tests were two. Results show a very high correlation of variant frequencies in PGx–CNS and European CEU population, as compared to African and Chinese populations.

To investigate multivariate patterns of variation among gene variants within the PGx–CNS sample (501 individuals), a nonlinear principal component analysis (PCA) was conducted using the 24 gene variants as variables [40–42]. Prior to this procedure, these categorical variables were binarized and subjected to the optimal scaling procedure. The output of this analysis was identical to that produced by a multiple correspondence analysis on the same data [43]. The number of principal components plotted was decided based on the standard scree-plot procedure [44]. All statistical analyses were performed in the IBM SPSS software package (IBM Inc., Armonk, NY; version 24 for Windows).

## Results

### Development of the iDNA PGx–CNS panel: Assessment and evaluation of the PGx targets

A robust *in-house* database (PGx–CNS database) for the pharmacogenetic analysis of multiple PGx associations was developed for each drug (Additional file 1, 2). Each association was selected on the basis of the available bibliographical evidence of clinical value, in line with the curation and validation method of the PharmGKB. The PGx–CNS database includes (a) the three possible combinations of the inherited genotypic variations for each target, i.e. homozygous wild type, heterozygous and homozygous mutated alleles; and (b) the PGx interpretation and advisory claim of each variant combination-drug association, for each drug. The available derived PGx information varies for each drug and depending on each gene–drug association, it comprises one or more of the following advisory categories: ‘Efficacy’, ‘Metabolism’, ‘Pharmacokinetics (PK)’ and ‘Toxicity’, indicating interpretations on drug response; clearance, metabolization and resistance; dosage; ADEs (miscellaneous); ADE (weight gain); ADE (metabolic syndrome); ADE (tardive dyskinesia); ADE (hypertriglyceridemia); ADE (hyperprolactinemia), ADE (gastroenteric) and ADE (stroke) (Additional file 1).

A bioinformatics platform was developed for the analysis of raw genotypic data from patients, their mapping to the PGx targets residing in the *in-house* database, the assignment of the gene variant-drug PGx information, and the report building and generation. The platform implements an algorithm which infers interpretations of the PGx results and provides as output a pdf report that contains all required information for clinical decision support, including all the variant-drug PGx information and respective PGx clinical advices as mentioned above. Essentially the algorithm makes use of evidence-based and published biological knowledge to assign a score to each drug of the PGx–CNS panel while considering the genetic variations found in the patients’ genotype. Based on the inferred score, drugs are reported to have a low, medium or high genotype-drug interaction. For drugs of high or medium genotype-drug interaction, clinical advices guiding the medical decisions, are provided. Drugs of low genotype-drug interaction are recommended to be administered according to the instructions provided in the drug label. The categorization considers the clinical significance scores of variant-drug associations and the significance scores of the biological function of each associated gene. More specifically, the significance scores of variant-drug associations are derived from the PharmGKB (can be 1A, 2A, 2B, 3) as shown in Additional file 1 [10]. An annotated variant-drug combination of Level 1A corresponds to a CPIC [45] or medical society-endorsed PGx guideline, or is found implemented at the Pharmacogenomics Research Network (PGRN) site, or in another major health system. An annotation of Level 1B refers to the preponderance of evidence, replicated in more than one cohort with significant p-values, and with a strong effect size. An annotation of Level 2A qualifies a VIP (very important pharmacogene) of functional significance, as defined by PharmGKB [10]. An annotation of Level 2B refers to moderately replicated evidence of association. A Level 3 annotation is based on a single significant (not yet replicated) study or multiple studies but lacking clear and conclusive evidence of an association. Finally, Level 4 annotations, based on a case report or a non-significant study, are not included in the panel presented here. In addition, those targets having guideline annotations by the FDA: US Food & Drug Administration, according to the FDA's “Table of Pharmacogenomic Biomarkers in Drug Labels’’ found on PharmGKB [10], are also noted (Additional file 1). The significance of the associated gene function is reflected by a prioritization grouping (PG) of the related genes that considers the biochemical significance of each encoded protein in its functional association with the drugs (metabolization or other effects). The prioritisation grouping is the following: Group A (high priority, score 3, including metabolising enzymes): CYP2C9, CYP2C19, CYP2D6, UGT2B7; Group B (intermediate priority, score 2, including signaling proteins): EPHX1, FKBP5, ANKK1/DRD2, DRD2, DRD3, HTR2C; Group C (limited priority, score 1, including additional signalling proteins): MC4R, GRIK1, SCN1A (Additional file 2).

Each genotype-drug association has been manually assigned to either ‘low, medium or high' genotype-drug interaction categories, corresponding to ‘minimum, intermediate or enhanced’ PGx effect. The implemented algorithm takes as input the patient’s genotype and calculates a drug score per category as the sum of the variant-drug significance and gene significance scores. More specifically, let *c* denote the genotype-drug interaction category which can be one of Low (*L*), Medium (*M*), High (*H*), *D* the set of genotype-drug associations relevant for the category examined, *EL* their PharmGKB evidence level, *G* the set of genes associated with the relevant genotypes and *PG* the prioritization group of the gene, assigned as mentioned above. Then, a drug is assigned to the category with the highest calculated score as follows:

$$ drug_{category} = argmax_{{c \in \left\{ {L,M,H} \right\}}} \left\{ {\sum\limits_{i \in D} {EL_{i} } + \sum\limits_{j \in G} {PG_{j} } } \right\} $$

In other words, the drug is assigned to the category (*L*, *M* or *H*) for which the total sum of the sum of the category related evidence levels and the sum of gene prioritization scores is maximized. Furthermore, to support the treatment decision by the clinical doctor, genotype-drug associations belonging to the categories of medium and high interaction are accompanied with a brief clinical advice. In total nine clinical advices have been defined and are provided as part of the PGx report.

In conclusion, the PGx–CNS constitutes an exhaustive set of clinically valuable markers and a prioritization analysis method for providing a robust PGx information on the selected 28 drugs, adequate for clinical usage.

## Validation samples and patient characteristics

**Fig. 1:**
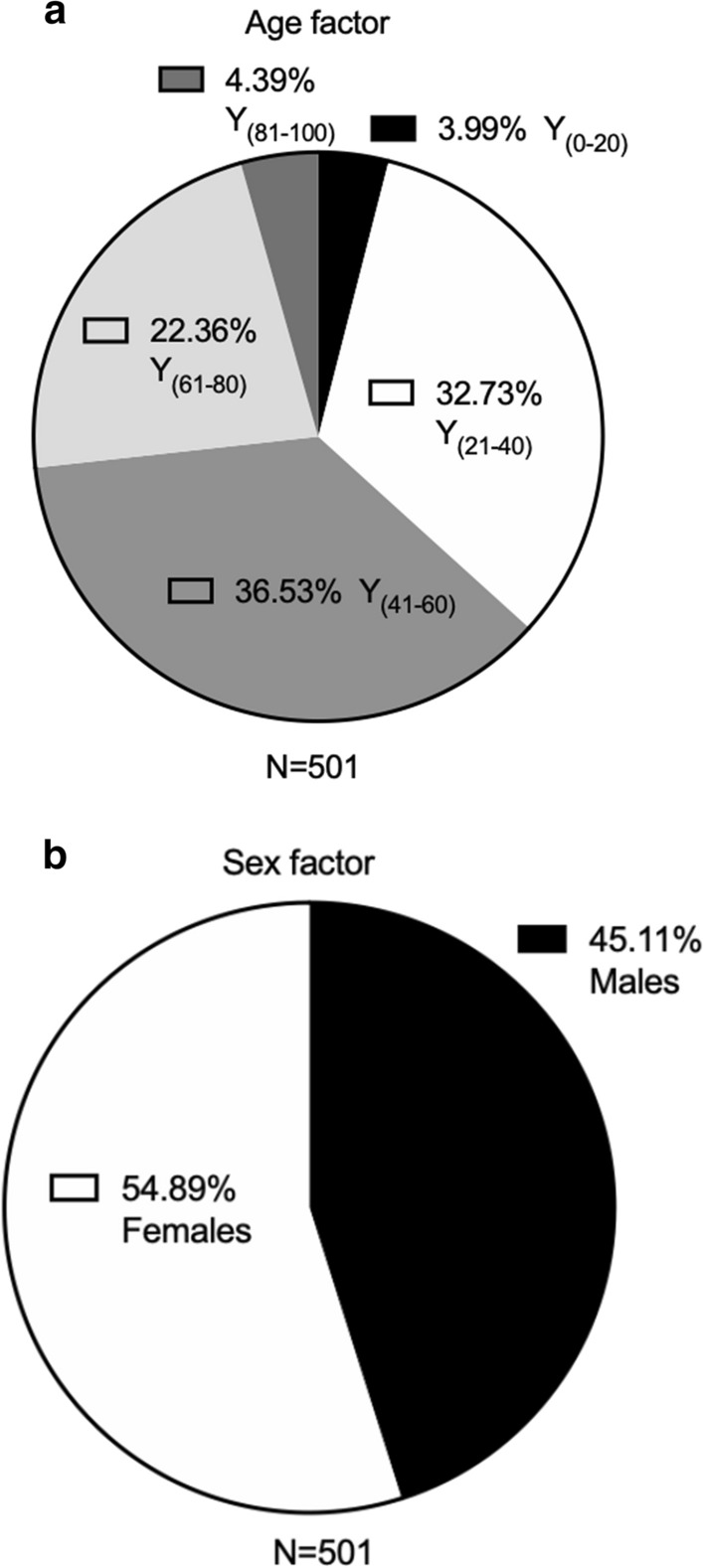
Pie chart of composition of 501 patient samples from southern Europe, in respect to the following parameters: **a** age factor in years (y) (0–20; 21–40; 41–60; 61–80; 81–100); and **b** sex factor (male or female)

Five hundred and one samples were collected from southeastern European individuals diagnosed with one or more conditions related to the CNS. The 501 samples constitute of 20 samples of 0–20 years old (4%); 164 samples of 21–40 years old (32.7%); 183 samples of 41–60 years old (36.5%); 112 samples of 61–80 years old (22.4%); and 22 samples of 81–100 years old (4.4%), (Fig. 1a; Additional file 4); corresponding to 226 males (45.1%) and 275 (54.9%) females (Fig. 1b; Additional file 4).

## Genotyping using the SNP TaqMan Real-time PCR method, provides with a quick and robust targeted determination of SNPs

All 501 patient-derived genomic DNA samples were genotyped with the SNP TaqMan Real-time PCR assay (Table 1; Additional file 3) showed an increased amplification efficiency, as the mean Ct for most of the targets was around 25; while the assay C_32407232_50 gave an amplification product at a Ct of 35, without affecting the final genotyping result. In order to confirm the validity and robustness of the followed methodology, we performed a comparative population analysis on the occurrences of each variant in different populations (Table 1; Additional file 3). Three populations were used for a comparative analysis with the 501 samples tested here, as shown in Table 1 (Additional file 3). The statistical results on 501 Southeastern European Caucasian (SEC) samples (PGx–CNS), show that the occurrences of each of the 24 variants are significantly similar to the population of European ancestry (CEU), compared to African (YRI) and Asian (CHB) populations (Table 1; Additional file 3). Indeed, after adjusting p-values using the Holm-Bonferroni sequential technique [39], the differences between PGx–CNS variant frequencies and CEU (European) were not found to be statistically significant (i.e., p-value > 0.05). However, 15 out of 24 differences between PGx–CNS and the African sample (YRI) were significant (p-value < 0.001, ***); both before and after adjusting p-values [39]. Based on the Cramer’s V coefficient, these differences were either moderate (V = 0.21–0.34) or strong (V ≥ 0.35) [37]. The strongest associations were observed for gene variants rs2832407 (0.73), rs963468 (0.49), and rs1414334 (0.48); while the variants not presenting significant results were rs4244285, rs4986893, rs17782313, rs28399504, rs35642686, rs5030655, and rs5030656. Comparing the PGx–CNS—Asian (CHB) samples, 14 out of 24 differences were significant (p < 0.01), eight of which also remained significant after Holm-Bonferoni adjustment. The strongest differences were for rs1065852 (0.35) and rs1800497 (0.32) (Table 1; Additional file 3). In conclusion, the results of the employed genotyping method are consistent with population studies and indicate that the variant frequencies of SEC individuals analysed here are statistically consistent to the European population; thus validating the employed analysis method.

**Fig. 2:**
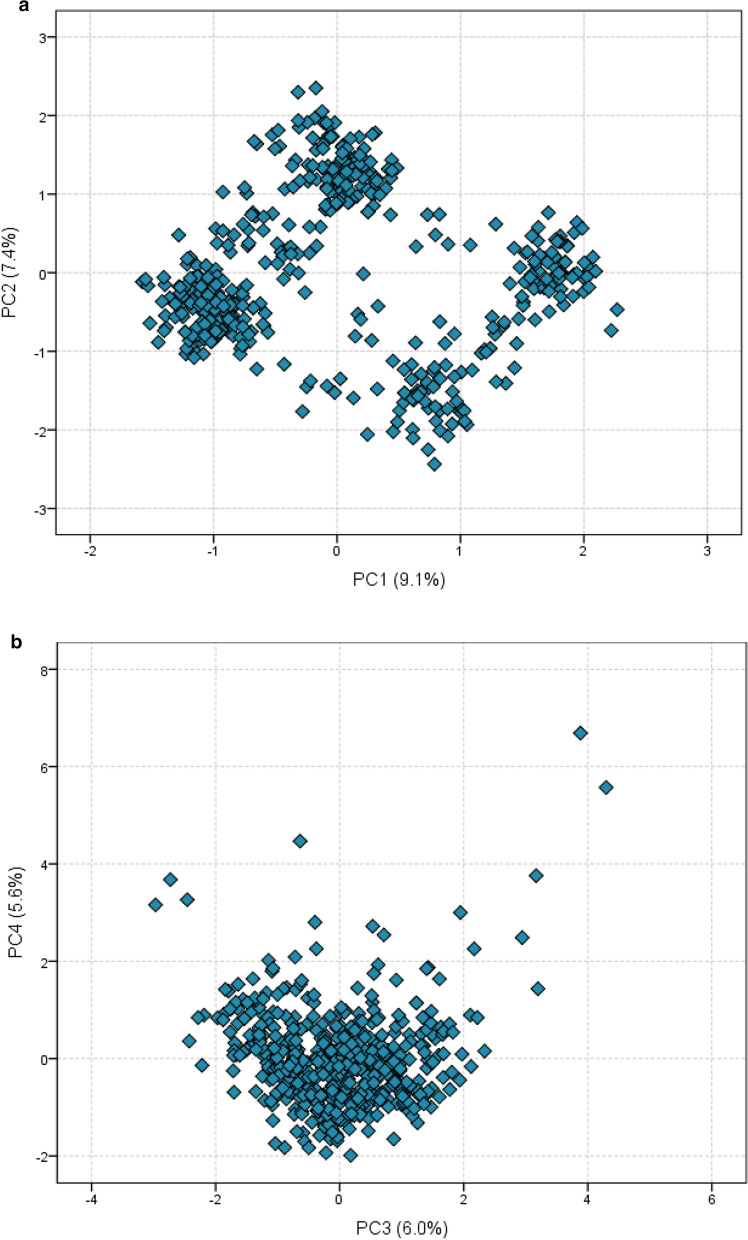
Nonlinear principal component analysis (PCA) on 24 gene variants used as variables, to investigate multivariate patterns of variation among the gene variants within the PGx–CNS sample (501 individuals). **a** PC1,2 (representing a partial amount of total sample variance of 28.01%) showed correlations among gene variants, distinctively forming four groups of individuals. **b** PC3,4 representing a partial amount of total sample variance of 11.6%, revealed the presence of two specimens with extreme scores (outliers)

In order to assess possible association/correlation profiles within the PGx–CNS sample, we ran a nonlinear PCA [40, 42] on the 24 pharmacogenetic variants (Fig. 2; Additional file 5), without assuming any grouping criterion for each of the 501 individuals. The scree-plot procedure recommended focusing on the first four PCs, which represent a partial amount of total sample variance (28.01%; Additional file 5). Results show that the combinations of the identified polymorphisms reveal the presence of four clusters in the sample, one for each direction of PC1 and PC2 (Fig. 2a). Based on the factor loadings (Additional file 5), individuals with positive scores on PC1 (9.1% of sample variance) tend to share the co-occurrence in genes encoding *UGT2B7* (rs7668258) and CYP2D6*41 (rs28371725), combined with the absence of *MC4R* (rs17782313, rs489693) and CYP2D6*4,*10 (rs3892097, rs1065852). Reversely, samples with negative PC1 values tend to reflect the co-occurrence of genes *MC4R* (rs17782313, rs489693) and CYP2D6*4,*10 (rs3892097, rs1065852), in combination with the absence of *UGT2B7* (rs7668258) and CYP2D6*41 (rs28371725). On the vertical axis PC2 (7.1%), individuals with positive values show a tendency to present *MC4R* (rs17782313 and rs489693) and the co-occurrence of genes encoding CYP2D6*4 (rs3892097) and CYP2D6*10 (rs1065852). The opposite tendency is observed in individuals with negative PC2 scores. On both PCs (16.5% of sample variance explained), the two variations of *MC4R* (rs17782313, rs489693) co-occur with an increased frequency for most individuals, indicating that they are interlinked (Fig. 2a). The distribution of specimen values on the remaining PCs (PC3 and PC4; 11.6% of total sample variance) reveals the presence of two specimens with extreme scores (i.e., individuals with unusual combinations; Fig. 2b; Additional file 5).

## Relevance of pharmacogenetic associations for CNS drugs: bioinformatic analysis and PGx interpretation

**Fig. 3:**
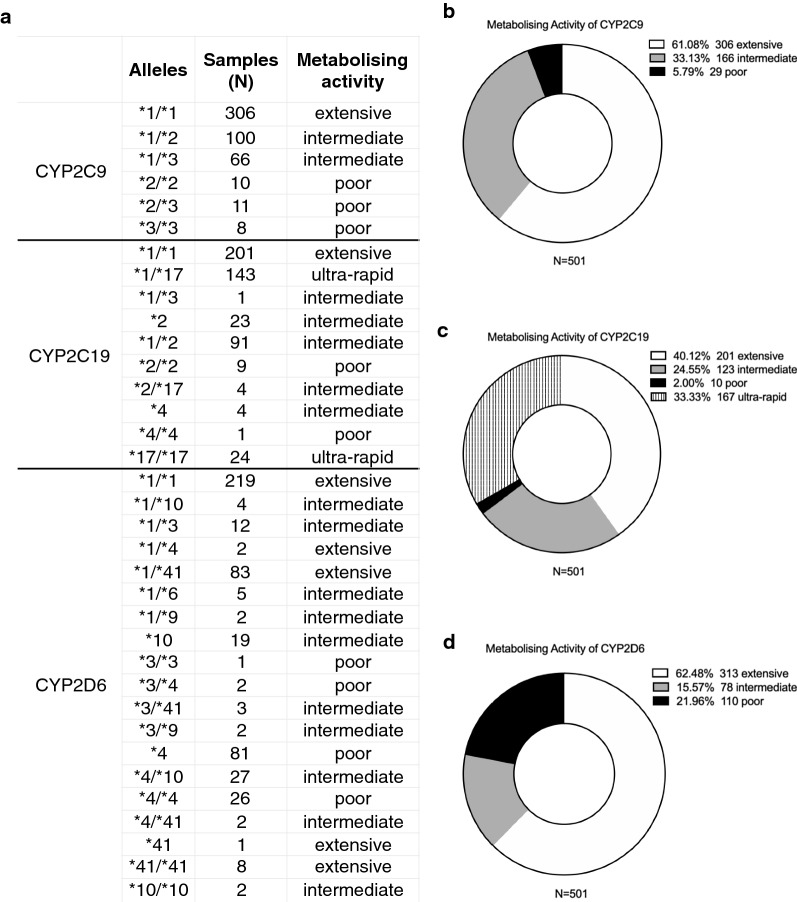
Analysis of the metabolisation activity of the cytochromes on 501 patients. **a** Reference table listing the categorisation of the allelic forms of each gene with the expected metabolisation efficiency as in ‘ultra-rapid’,’extensive’, ‘intermediate’ and ‘poor’. **b, c, d** Pie chart of the percentile distribution of the metabolising activity of CYP2C9 (**b**), CYP2C19 (**c**) and CYP2D6 (**d**). Results indicate that 40% to 60% of patients carry at least one CYP gene with altered metabolising efficiency

Genotypic results targeting the 24 variants from 501 samples, were analysed by the developed iDNA-PGx–CNS bioinformatic platform, which allows the automation of the PGx interpretation procedure from raw genotypic data. The analysis involves the assignment of the drugs comprising the PGx–CNS panel to either low, medium or high genotype-drug interaction categories per patient, corresponding to ‘minimum, intermediate or enhanced’ PGx effect. Results showed the distribution of the metabolisation activity of the cytochromes (CYP2C9, CYP2C19 and CYP2D6), categorised as ultra-rapid, rapid/extensive, intermediate or poor metabolisers (Fig. 3; Additional file 6). Homozygote star alleles *2 and *3 of *CYP2C9* and of *CYP2C19* or their combination, were considered as poor metabolisers, while heterozygotic combinations of *1 or *17 are considered as intermediate metabolisers. The *17 of *CYP2C19* was considered as an ultrarapid metabolizer [30, 46] (Fig. 3a). The allelic combinations *4/*10 and *4/*41, *1/*9, *1/*10, *1/*3, *1/*6, *3/*41, *3/*9 of *CYP2D6*, were considered as intermediate metabolisers, while the *3/*3, *3/*4, *4/*4, as poor metabolizers. The combinations *1/*1, *1/*4, *1/*41 and *41/*41 were considered as extensive metabolisers [46] (Fig. 3a). Results also showed that for *CYP2C9*, 306 samples (61%) indicate rapid/extensive metabolisers; 166 (33%) indicate intermediate; and 29 (6%) indicate poor metabolisers (Fig. 3b). For *CYP2C19*, the values are 201 (40%); 123 (25%) and 10 (2%), respectively, while 167 (33%) are ultra rapid metabolisers (Fig. 3c). For *CYPD6*, the values are 313 (62%); 78 (16%) and 110 (22%) respectively (Fig. 3d). Overall, results indicate that 40% to 60% of the tested patients carry at least one CYP gene with altered metabolising efficiency, with valuable PGx information.

**Fig. 4:**
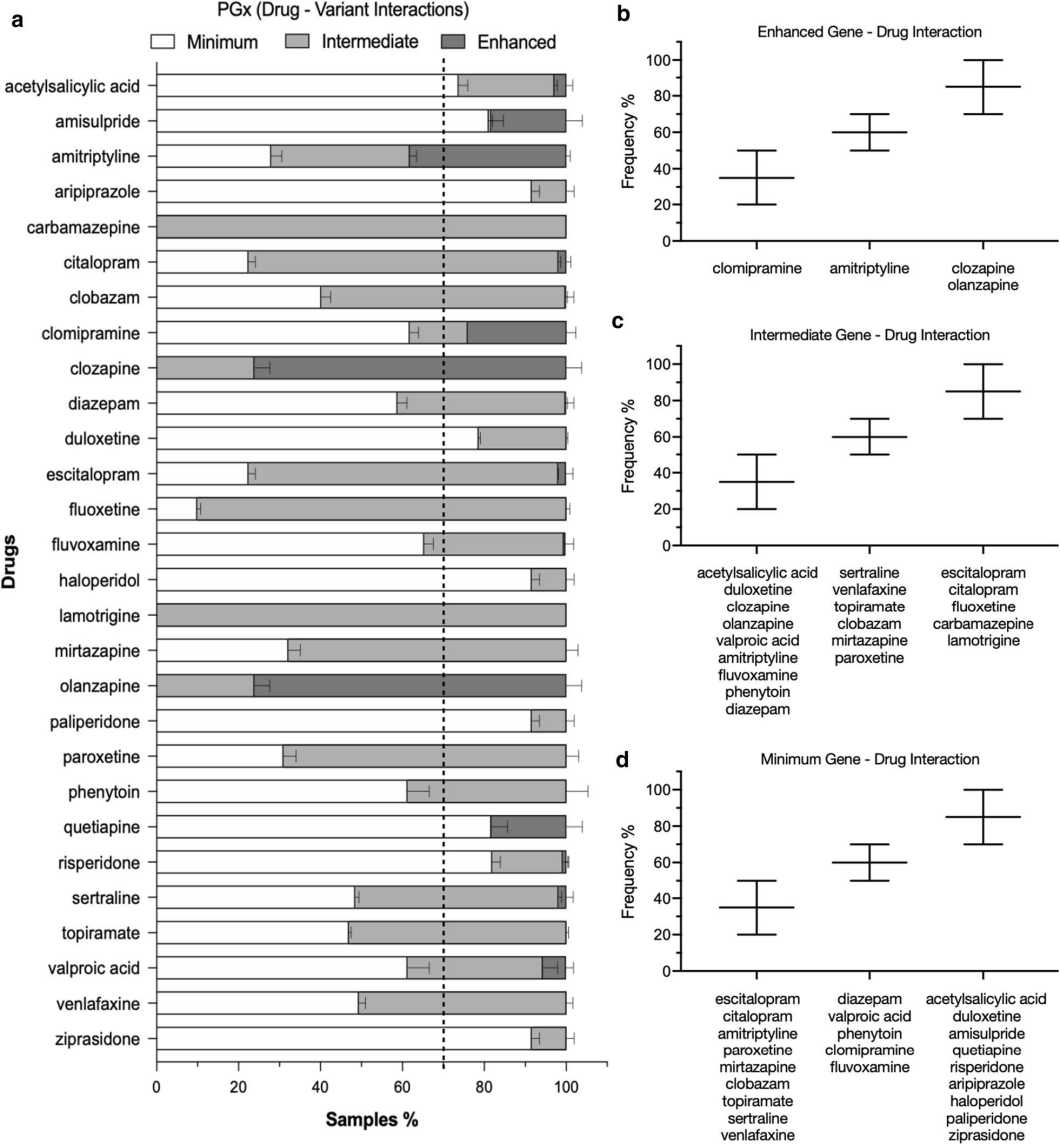
Statistical analysis of the drugs associated in gene-drug interactions and PGx information, on 501 patients. Results are indicative of the drug relevance in the sample, based on the frequencies of gene-drug interactions, in the southeastern European population. **a** Frequency % of gene-drug interactions per drug, deriving from statistical analysis on 501 samples. The deviation bars denote the variations between male and female samples. White: minimum gene-drug interaction; light grey: intermediate gene-drug interaction and dark grey: enhanced gene-drug interaction. **b** Frequency % of drugs exhibiting enhanced gene-drug interactions (20–100%). **c** Frequency % of drugs exhibiting intermediate gene-drug interactions (20–100%). **d** Frequency % of drugs exhibiting minimum gene-drug interactions (20–100%)

In order to identify the PGx associations with increased occurrences (N = 501), we performed a statistical analysis of the PGx information deriving from the gene-drug associations (Fig. 4a; Additional file 4). Results show a variation in the PGx associations, linked to clinically relevant information that may be used in medical decision. Overall, a subset of the targeted 28 drugs may be proposed as statistically more relevant to the SEC population, as the frequency of polymorphisms indicating ‘therapeutically actionable PGx associations’, for which genetic testing is recommended, is high (Fig. 4). In fact, more than 70% of the samples (patients) correspond to enhanced gene-drug interactions, for clozapine and olanzapine (Fig. 4b), and intermediate gene-drug interactions for the drugs escitalopram, citalopram, fluoxetine, carbamazepine and lamotrigine (Fig. 4c). On the contrary, the following drugs exhibited minimum gene-drug interactions at a high percentage (> 70%): acetylsalicylic acid, duloxetine, amisulpride, quetiapine, risperidone, aripiprazole, haloperidol, paliperidone and ziprasidone (Fig. 4d). This indicates a high occurrence of PGx associations for these drugs and an increased relevance of a genetic test preceding the medical therapeutic decision.

## Clinical evidence

The PGx analysis is a crucial medical tool in the hands of any clinician in order to help patients get more effective and safer treatments while avoiding the “*trial and error*” strategy. Here we describe four cases, provided as indicative examples of patients whose treatment was modified by the physician, on the basis of their PGx–CNS results.

## Case a: pervasive developmental disorder not otherwise specified

The first case is a 19-year- old Caucasian male with a medical history of pervasive developmental disorder not otherwise specified (ICD-10-CM Code F84.9).^4^ During the last two years he took several drug combinations (mostly antipsychotics and mood stabilizers) to control hostility, aggressiveness and psychomotor anxiety with poor effectiveness. The PGx–CNS analysis gave interesting information regarding the use of psychotropic medications. The *DRD2* gene (rs1799978, TT) is correlated with good response to risperidone treatment; and the *ANKK1/DRD2* (rs1800497, GG) is correlated with reduced risk for hyperprolactinemia and weight gain but increased possibility for tardive dyskinesia. The *CYP2C19* gene (rs4244285, AA indicating the *2/*2 genotype) is correlated with very slow metabolizing activity for amitriptyline, citalopram, escitalopram and sertraline. These antidepressants possibly require small doses and slow titration in this patient. In line with the PGx–CNS results, the patient subsequently received a treatment with olanzapine (10 mg/day), zuclopenthixol (20 mg/day) and diazepam (10 mg/day) and after 4 weeks, responded well. Aggression and anxiety levels were decreased, and the patient felt better. No serious adverse effects have been reported.

## Case b: paranoid schizophrenia

The second case is a 30-year old Caucasian female with a medical history of paranoid schizophrenia (ICD-10-CM Code F20.0)^5^ Previous antipsychotics treatments were correlated with adverse effects (hyperprolactinemia with risperidone, weight gain with olanzapine and severe sedation and hypotension with quetiapine). Due to these adverse effects the compliance was poor and the paranoid symptoms persist. The PGx–CNS analysis gave information that other antipsychotics like aripiprazole and haloperidol could possibly be more suitable regarding her ADEs. The *HTR2C* gene (rs1414334, CG) and the *MC4R* gene (rs17782313, CT) are correlated with an increased risk of developing metabolic syndrome and weight gain in this patient. In line with the PGx–CNS results, the patient subsequently received a treatment with haloperidol (10 mg/day) and aripiprazole (15 mg/day) and after 4 weeks, responded well. Psychotic symptoms were decreased and the patient felt relief with enhanced functional levels. No serious adverse effects have been reported.

## Case c: mixed anxiety and depressive disorder

The third case is a 56-year- old Caucasian female with a medical history of mixed anxiety and depressive disorder (ICD-10-CM Code F41.2). She has never reached a remission state with several antidepressant treatments (monotherapy and combinations). Due to adverse effects in the first days of treatment she reported discontinuation for some antidepressants and especially for selective serotonin reuptake inhibitors (SSRIs). Residual symptoms like anhedonia, concentration difficulties and anxiety still persist. The PGx–CNS analysis gave information regarding the use of psychotropic medications in this patient. The *CYP2C19* gene (rs4244285, AG; rs28399504, AA and rs12248560, CT) is correlated with possibly slow metabolizing activity for antidepressants like citalopram and escitalopram. The *FKBP5* gene (rs4713916, GG) is correlated with possibly lower response rates in many antidepressants like fluoxetine, paroxetine and sertraline in this patient. In line with the PGx–CNS results, the patient subsequently received a treatment with duloxetine (60 mg/day) and after 4 weeks, responded well. Depression and anxiety symptoms were decreased and the patient felt better. No serious adverse effects have been reported.

## Case d: recurrent depression

The fourth case is a 49-year- old Caucasian male with a medical history of recurrent depression (ICD-10-CM Code F33.2)^6^ The main issue in this patient was remission after many years of antidepressants treatments with moderate results. The PGx–CNS analysis gave interesting information regarding the use of psychotropic medications in this patient. The *CYP2C19* gene (rs4244285, AG; rs4986893, GG; rs28399504 AA and rs12248560, CC) is correlated with possibly slow metabolizing activity for citalopram, escitalopram, sertraline, amitriptyline and clomipramine. The *CYP2D6* gene (rs3892097, CT and rs1065852, AG) is possibly correlated with a slow clearance (and a high possibility of adverse effects) for paroxetine and mirtazapine. On the other hand, the *FKBP5* gene (rs4713916, AA) is possibly correlated with good response rates for antidepressants like fluoxetine and venlafaxine in this patient. In line with the PGx-CNS results, the patient subsequently received a treatment with fluoxetine (20 mg/day) and duloxetine (60 mg/day) and after 4 weeks, responded well. Depression symptoms decreased and the patient felt better. No serious adverse effects have been reported.

## Discussion

We introduced a pharmacogenetic panel (iDNA Genomics-PGx–CNS or PGx–CNS), consisting of 24 SNPs on 13 genes that can be used to optimize the selection of the treatment and support medical decisions for diseases of the CNS, by providing individualized pharmacogenetic information about the response, the efficacy of the treatment and the ADEs of 28 drugs. Patients’ genotyping results are interpreted into pharmacogenetic associations with phenotypic impacts of clinical interest, and are presented in the form of reports describing clinical guidelines in drug selection and dosage. Importantly, the PGx information of the presented panel of 24 variants is based on curated information from knowledge bases, such as PharmGKB. Targets with adequate evidence were exclusively selected to be included in the pharmacogenetic panel. It is noted that the analysis of *CYP2D6* copy number variations (CNVs), which is needed to properly determine patients’ phenotype and the choice of the correct dose of drugs affected by CYP2D6 in normal clinical practice [47], was not investigated in this study as such an analysis requires different experimental settings and approaches such as Next Generation Sequencing or long range PCR [48]. More specifically, previous research has shown that 12.6% of the general US population have CNVs in the *CYP2D6* gene, with certain potential quantitative, rather qualitative interpretative consequences, as compared to the activity of the duplicated allele. This is indicated in cases where a duplication of a normal allele or an allele of increased activity, may lead to an overall increased metabolising activity [49]. In addition, with respect to Carbamazepine (CBZ) this study focuses on *EPHX1* and *SCN1A* genes, but does not test PGx polymorphisms on the HLA gene (HLA-A and HLA-B alleles), associated with increased risk of CBZ-induced hypersensitivity syndromes, with diverse distribution in different populations [50]. Patients who test positive for HLA-A*31:01 can be prescribed alternative anti-epileptic drug therapy, such as lamotrigine, which has not been associated with hypersensitivity in HLA-A*31:01 carriers, while in patients positive for HLA-A*31:01 who still require CBZ therapy, PGx testing may still help alert clinicians to monitor these patients more closely [51]. Overall, the proposed PGx–CNS analysis can be used by psychiatrists and neurologists as a decision support evidence-based test, for the treatment of CNS diseases. It is provided as a service by the iDNA Genomics Private Company.

Our results show the validity of the test in respect to the genotypic analysis and the clinical pharmacogenetic interpretation. The SNP TaqMan Real-time PCR method is shown as a rapid ideal method to perform genotyping variant determination. It is a *state-of-the-art* robust, highly reproducible approach for determining known variations in the DNA, by employing a set of 2 primers and 2 probes (fluorophores FAM or VIC), selectively and interchangeably hybridizing with the DNA sequence, depending on the presence or absence of the variation. The raw data also contain real time measurements of the amplification product(s). All the genotyping results were based on high amplification efficiencies. However, in comparison to the mean Ct for most of the targets (around 25), the assay C_32407232_50, in certain samples had a Ct of 35. This indicates that it had a quantitatively (but not qualitatively) decreased efficiency in hybridising, resulting to a lower Ct. This is a rare event probably due to amplification interference caused by SNPs occurring on the hybridizing sequences of the primers or the probes, which may well vary on populations [52]. In cases the interference is high, it can be avoided by employing alternative assays (primers and probes) targeting the variation. Allele drop-out due to neighbouring SNPs, may conceivably occur in any assay amplifying relatively short PCR products and is not limited to TaqMan technology [52]. Yet, this quantitative discrepancy on the Ct value reflects on the quantitative rather the qualitative outcome and does not alter the final result.

The genotyping results from 501 patients were used in a statistical analysis. To our knowledge, it is the first time the selected PGx variants are analysed together in the frame of a single panel, on samples derived from Southeastern European patients. Their frequencies were compared to three populations, aiming to evaluate the overall methodology and robustness of the results. As expected, the genotyping variation frequencies exhibit a close similarity to European populations, compared to Chinese and African populations. The cohort of 501 patients with different neurological or/and psychiatric conditions, represents a selected neurological or psychiatric patient group, which may present variations compared to randomised SEC populations. It would be of interest to compare the obtained results with genomic data from randomised Greek and SEC populations, to address whether the frequencies identified here, are linked to neurological or/and psychiatric conditions. For example, results indicate that 40% to 60% of the patients carry at least one *CYP* gene with an altered metabolising activity, but it is not known whether these frequencies are constitutively linked to predisposition. However, as expected, the occurrences of the targeted polymorphisms in the patient-derived samples, are closer to the European population, compared to Asian or African populations, thus validating the followed methodology (Fig. 2). This is in line with studies using microarray analyses indicating a close relationship between European and south European populations [53]. As a result, these findings confirm the validity of the employed analysis method. Interestingly, the present study’s nonlinear PCA on the 501 samples showed distinctive variation among four groups of individuals, corresponding to different correlations among gene variants (Fig. 2a; Additional file 5). One of the observed patterns involved the frequent co-occurrence of the rs17782313 and rs489693 variants on the *MC4R* gene, implying that targeting one of these variants in some individuals may sufficiently address the clinically interesting pharmacogenetic associations of the *MC4R* gene. In some samples, variations on *MC4R* gene (rs17782313, rs489693) tended to co-occur with CYP2D6*4,*10 (rs3892097, rs1065852), but with no variations on rs7668258 (*UGT2B7* gene). In fact, variation on rs7668258 (*UGT2B7* gene) tends to co-occur with CYP2D6*41 (rs28371725 variation). These results point towards a higher relevance of combined CYP2D6*4,*10 and *MC4R* variations as well as a correlation of CYP2D6*41 with *UGT2B7* variations. This is in line with previous data showing a general combined co-occurence of *CYP2D6* / *UGT2B7* variability [54].

In addition to the presented statistical findings on the variants, the selected gene-drug associations, of the PGx–CNS panel, were used to study the relevance of the presented drugs in the population (ie frequency of enhanced PGx associations), aiming to address their high translational value. This task required the interpretation of the genotypic results to PGx associations according to the literature, and importantly, the combination of the PGx results, by an algorithm accounting the EL and PG scores. Indeed, depending on each drug and according to the described algorithm, the PGx results derive from a combination of PGx associations of subsets of the 24 polymorphisms. Each drug presents a different number of associations of clinical value. As an example, the PGx–CNS panel includes the analysis of 6 variants corresponding to five genes (*CYP2D6, ANKK1/DRD2, DRD2, HTR2C, MC4R*) for risperidone and one variant (*FKBP5* gene), for fluoxetine (Additional file 1). The combination algorithm is based on a score-ranking method whereby the EL and PG scores determine the final interpretation to provide with an overall conclusion about the pharmacogenetic compatibility of the patient with the each drug (Additional file 1). It becomes clear that including additional markers in the panel (clinically useful PGx associations) of poor EL or PG scores according to the current literature, would not alter the conclusive drug-specific interpretation as the resulted scores would be relatively low. Thereof, the selected 24 markers of the PGx–CNS panel adequately support a conclusive PGx reporting for the clinically valuable PGx analysis of the targeted drugs.

The described drug relevance in the population, highlights a subset of drugs, which are more commonly associated with polymorphisms denoting an altered activity linked to PGx information (ie metabolization activity, Fig. 4). The metabolisation activity of three genes of the P450 (relevant to the targeted drugs), showed that 61% of the samples were rapid/extensive metabolisers for CYP2C9, 33% were intermediate, and 6% were poor metabolisers (Fig. 3b). These results are consistent with previous findings on the distribution of metabolising activity based on the variant occurrences in Ashkenazi Jewish (Mediterranean), Caucasian and other populations [55, 56]. For CYP2C19, the metabolising activities were 40% (extensive), 25% (intermediate), 2% (poor), and 33% (ultra rapid) metabolisers. These results are in line with previous findings on the distribution of variants in randomised American and in Russian patients [57, 58], (Fig. 3c). For CYPD6, the values were 62% (extensive), 16% (intermediate) and 22% (poor). These results are consistent with previous findings on the distribution of single copy variants in the United States [59]. These results indicate a high percentage of occurrences of individuals carrying variants associated with poor or intermediate metabolization activity of the cytochromes (Fig. 3), urging the implementation of similar PGx testing in additional drugs employed for the treatment of other diseases [60–62]. Accordingly, > 70% of the individuals (patients) exhibited intermediate or enhanced gene-drug interactions for a range of CNS drugs (escitalopram, citalopram, fluoxetine, carbamazepine and lamotrigine; clozapine and olanzapine) (Fig. 4b, c), urging the application of the PGx–CNS test for the treatment of psychotic and neurological diseases. However, these analyses reflect bio-statistical observations based on the frequencies of the involved polymorphisms on a cohort of patients with different neurological or/and psychiatric conditions, and thus it may not independently signify therapeutic medical choices. It should be highlighted that the selection of the therapeutic strategy derives from multifaceted medical decisions, applied by the physician, who takes into consideration various genetic, clinical and environmental factors, which may influence drug efficacy. The disease status and the disease itself may influence the fate of the drug in vivo*,* by altering the phenotype of a certain genetic trait. Interestingly, from the genetic viewpoint the sex factor did not show a high disparity in the occurrence of the variants (higher than 10%, Additional file 4). However, it is noted that the data for valproic acid (VPA) and phenytoin exhibited a deviation of 5.4% between Males and Female individuals, corresponding to a higher frequency of minimum gene-drug interactions in Males (Additional file 4: the sex factor). This is in line with studies showing that Age and Sex factors influence VPA serum concentrations, and older female patients generally required 30–50% lower dosing of VPA compared to younger males [33]. Such deviations in the PGx outcome may indeed be influenced by the hormonal status, the metabolism and the age of the patients. Finally, the described clinical cases randomly selected from the pool of the 501 samples by the clinicians, show that patients have benefited from a PGx–CNS—guided treatment, by increasing their response and eliminating the ADEs. In conclusion, implementation of PGx analysis on the presented 28 CNS drugs offers an additive knowledge in a patient-based personalized treatment.

## Conclusions

In conclusion, this work presents a pioneer PGx panel consisting of 24 targets, adequately identifying the PGx profile of individuals (patients) for 28 CNS drugs. The results from 501 CNS patients overall show that this pharmacogenetic analysis as a companion diagnostic assay preceding the therapeutic medical decision, is statistically relevant and of high importance for clinical practices. These results indicate that the described panel can be used as a standardised tool for testing PGx genes and alleles across clinical laboratories, providing recommendations as a reference guide for therapeutic selection for CNS conditions.

## Supplementary Information

**Additional file 1**: Drug-variant associations of the PGx–CNS panel. The PGx–CNS panel includes 28 CNS drugs and to 24 single nucleotide polymorphisms (SNPs). The table summarizes the SNPs and the genes associated to each of the 28 drugs. The corresponding probe IDs and the Evidence Levels (EL) are mentioned. The Level of evidence (EL) of each PGx variant-drug association, adequate for clinical use, in line with PharmGKB. The PGx-CNS panel includes pharmacogenetics associations of clinical significance. Each association has a variable level of evidence (EL), as derived by literature mining, in line with the rating system of "Strength of Evidence” by the PharmGKB [10]. In addition, the associations annotated by the FDA are noted (Actionable: The label may contain information about changes in efficacy, dosage, metabolism or toxicity due to gene/protein/chromosomal variants or phenotypes (e.g. "poor metabolizers"). Or the label may mention contraindication of the drug in a particular subset of patients with particular variants/genotypes/phenotypes. However, the label does not require or recommend gene, protein or chromosomal testing; Informative PGx: The label contains information stating that particular gene/protein/chromosomal variants or metabolizer phenotypes do not affect a drug’s efficacy, dosage, metabolism or toxicity, or its effect is not considered as “clinically” significant, or it does not currently meet the requirements to be assigned as “Actionable PGx”, No recommendation: The PGx association is not mentioned in the FDA's Table of Pharmacogenomic Biomarkers in Drug Labels).

**Additional file 2**: Functional roles of the genes associated with the PGx–CNS panel. The PGx–CNS panel includes 13 genes that were selected for their valuable PGx association with the targeted drugs. The table summarizes the functional roles of their encoded proteins, ranging from metabolization to cell signalling and actionable mechanisms [63–90].

**Additional file 3**: Genotypic results (tab1), SNP frequencies (tab2) and comparative population analysis on the occurrences of 24 variants identified on 501 patients (PGx–CNS) and three different populations (CEU, YRI, CHB) (tab3).

**Additional file 4**: Categorization of identified PGx associations of 28 drugs with 24 SNPs, on 501 patients as minimum (homozygotic, no polymorphism); intermediate (heterozygotic polymorphism) and enhanced (homozygotic polymorphism), including the Age and the sex factors.

**Additional file 5**: Statistics of the nonlinear principal component analysis (PCA) involving 24 variables (gene variants) and all 501 samples of the PGx–CNS panel.

**Additional file 6**: Categorization of identified PGx associations denoting altered metabolisation efficiency of CYP2C9, CYP2C19 and CYP2D6, on 501 patients, denoted as minimum (homozygotic, no polymorphism); intermediate (heterozygotic polymorphism) and enhanced (homozygotic polymorphism), including the Age and the sex factors.

## Data Availability

The data that support the findings of this study are available from iDNA Genomics but restrictions apply to the availability of these data, which were used under license for the current study, and so are not publicly available. Data are however available from the authors upon reasonable request and with permission of iDNA Genomics.

## Abbreviations

ADE: Adverse drug effect
CBZ: Carbamazepine
CEU: Utah Residents (CEPH) with Northern and Western European Ancestry
CEPH: Clinical epidemiology and population health
CHB: Chinese han in Beijing
CNS: Central nervous system
CNV: Copy number variation
FDA: US Food & Drug Administration
PCA: Principal component analysis
PGx: Pharmacogenomics
SEC: Southeastern European Caucasian
SSRIs: Selective serotonin reuptake inhibitors
SNP: Single nucleotide polymorphism
VIP: Very important pharmacogene
VPA: Valproic acid
YRI: Yoruba in Ibadan

## Footnotes

1 www.pharmgkb.org/page/cyp2d6RefMaterials.

2 www.pharmgkb.org/page/cyp2c9RefMaterials.

3 www.pharmgkb.org/page/cyp2c19RefMaterials.

4 Medical classification as listed by WHO under the range—Mental, Behavioral and Neurodevelopmental disorders.

5 Medical classification as listed by WHO under the range—Mental, Behavioral and Neurodevelopmental disorders.

6 Medical classification as listed by WHO under the range—Mental, Behavioral and Neurodevelopmental disorders.

## Declarations

### Study limitations

As discussed in the manuscript, the analysis of CYP2D6 copy number variations (CNVs) and PGx polymorphisms on the HLA gene, are not investigated in this study.
